# Estrogen enhances mismatch repair by induction of MLH1 expression via estrogen receptor-β

**DOI:** 10.18632/oncotarget.16351

**Published:** 2017-03-18

**Authors:** Jun-Yu Lu, Peng Jin, Wei Gao, De-Zhi Wang, Jian-Qiu Sheng

**Affiliations:** ^1^ Third Military Medical University, Chongqing, China, 400038; ^2^ Department of Gastroenterology, PLA Army General Hospital, Beijing, China, 100700; ^3^ Department of Gastroenterology, The First Affiliated Hospital of Dalian Medical University, Dalian, China, 116044

**Keywords:** colorectal cancer, estrogen, estrogen receptor beta, mismatch repair, MLH1

## Abstract

Epidemiological data demonstrated that hormone replace treatment has protective effect against colorectal cancer (CRC). Our previous studies showed that this effect may be associated with DNA mismatch repair. This study aims to investigate the mechanism of estrogen induction of MLH1, and whether colorectal tumor proliferation can be inhibited through induction of MLH1 by estrogen signal pathway. Human CRC cell lines were used to examine the regulation of MLH1 expression by over-expression and depletion of estrogen receptor-α (ERα) and estrogen receptor-β (ERβ), under the treatment with 17β-estradiol or β-Estradiol 6-(O-carboxy-methyl)oxime:BSA, followed by a real-time Q-PCR and Western blotting analysis. Luciferase reporter and chromatin immunoprecipitation assays were used to identify the estrogen response elements in the proximal promoter of *MLH1* gene. Then, the influence of estrogen-induced MLH1 on CRC tumor growth were determined *in vitro* and *in vivo*. We found that mismatch repair ability and microsatellite stability of cells were enhanced by estrogen via induction of MLH1 expression, which was mediated by ERβ, through a transcriptional activation process. Furthermore, we identified that ERβ exerted an inhibitory effect on CRC tumor proliferation *in vitro* and *in vivo*, combined with 5-FU, through up-regulation of MLH1 expression. Finally, we concluded that estrogen enhances mismatch repair ability and tumor inhibition effect *in vitro* and *in vivo*, via induction of MLH1 expression mediated by ERβ.

## INTRODUCTION

Estrogen has been demonstrated as a protective factor for colorectal cancers (CRC). Several studies showed that women are less susceptible than men, within same age group, to colon cancer [1–4]. Large-scale population analysis indicated that hormone-replacement therapy had a protective effect for postmenopausal women [5–7]. In our previous studies, we found that the risk of colorectal cancer was higher in males with mutated mismatch repair (MMR) genes than that in females with same mutations [8–10].

MMR genes encode a set of proteins to maintain genomic stability during DNA duplication, and defective MMR is closely related to the many malignancies, like Lynch syndrome. It's reported that mutations of *MLH1* and *MSH2* were the most common pathogenic genes in Lynch syndrome. Interestingly, the frequency of mutation and loss of expression of MLH1 was reported to be higher than that of MSH2 [8, 11–17]. Our group previously observed that the expression of MLH1 in colonic epithelial cells positively correlated with serum estrogen concentration (17β-estradiol > 45 pg/ml) [18], and treatment with estrogen up-regulated the expression of MLH1 *in vitro* [19].

However, the mechanism of estrogen-induced expression of MLH1 remains unclear. In this study, we investigated the molecular mechanism and found that ERβ significantly increased MLH1 expression in cells under the treatment with estrogen, by binding a specific region at *MLH1* gene promoter. And by this way, ERβ exerted anti-CRC effect *in vitro* and *in vivo*.

## RESULTS

### Estrogen enhances MLH1 expression through estrogen receptor pathway

**Figure 1:**
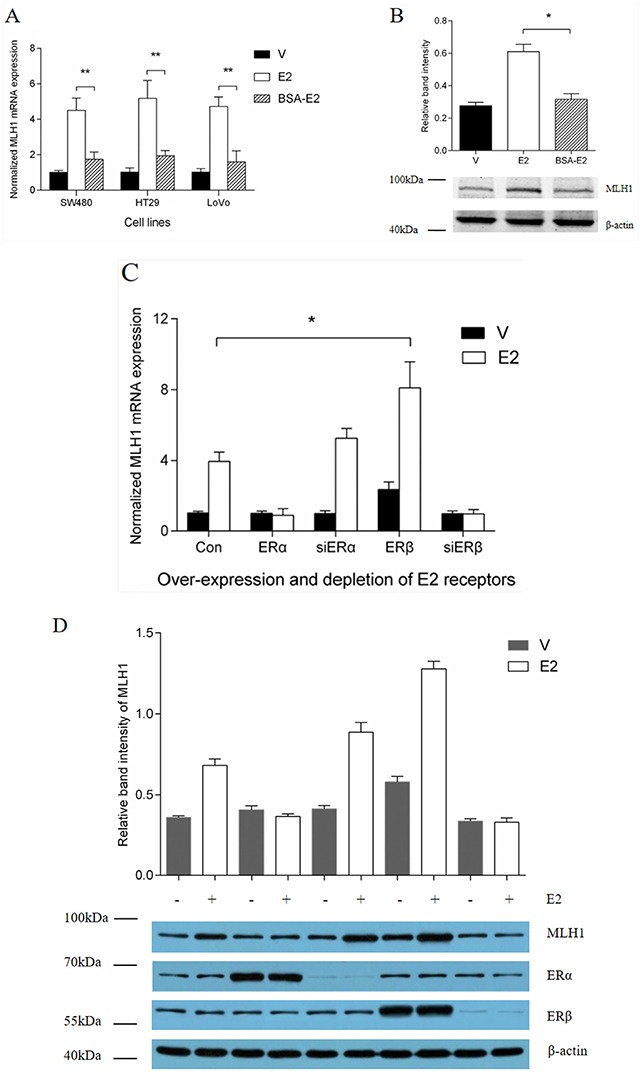

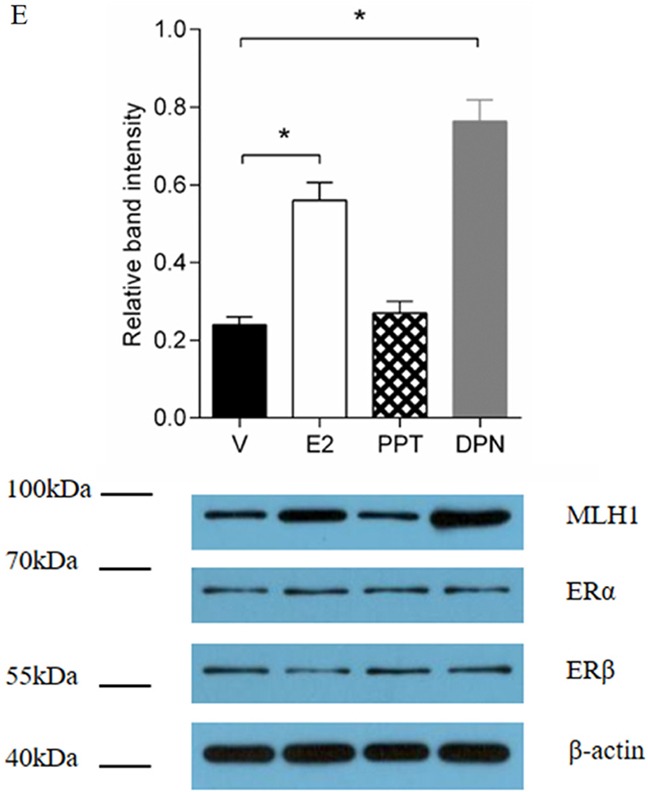
Effect of ERβ on estrogen induction of MLH1 expression **(A)** Normalized *MLH1* mRNA expression in SW480, HT29 and LoVo cell lines. Hormone-depleted cells in six-well plates were treated with vehicle, 10 nM E2, or BSA-E2 for 12 h. Total RNA were extracted and expression of *MLH1* was analyzed by Q-PCR. Values represent the mean ± S.D. (n=3). ** *p* < 0.01. **(B)** Hormone-depleted HT29 cells in six-well plates were treated with 10 nM E2, or BSA-E2 respectively. After 24 h, total protein extracts were analyzed by Western blotting. **(C)** Normalized *MLH1* mRNA expression in LoVo cells. Hormone-depleted cells in six-well plates were transient-transfected with ERα, ERβ expression or siERα, siERβ plasmids and empty control vector, respectively. At 24 h post-transfection, cells were treated with vehicle or 10 nM E2 for 12 h. Then total RNA were extracted and analyzed by Q-PCR. Values represent the mean ± S.D. (n=3). * *p* < 0.05. **(D)** MLH1 protein expression assay. LoVo cells were treated as part C, then ERα, ERβ and MLH1 expression level were detected by Western blotting. **(E)** MLH1 protein expression assay. LoVo cells in six-well plates were hormone-depleted, then treated with 10 nM PPT, E2, DPN and Vehicle, respectively. 24 h later, total protein were extracted and analyzed by Western blotting. Values represent the mean ± S.D. (n=3). * *p* < 0.05. E2 = Estradiol, V = Vehicle.

To reveal the mechanism by which E2 up-regulates the expression of MLH1, we treated the cells with two forms of E2, free E2 and BSA conjugated E2 in colon cancer cell lines. A real-time Q-PCR analysis showed that, E2 enhanced the *MLH1* gene expression significantly in all the three cell lines (Figure 1A, open columns), however, BSA-E2 showed very weak effect on the gene expression (Figure 1A, striated columns). A Western blotting analysis further indicated that E2 treatment greatly increased the protein level of MLH1 in HT29 cells (Figure 1B). These results suggest that E2 enhanced the expression of MLH1 both at mRNA and protein levels. Since BSA-conjugated E2 has less effect on the expression of MLH1, we can infer that E2 function on the regulation of the gene expression through typical estrogen receptor pathway.

### ERβ promotes MLH1 expression induced by estrogen

E2 binds to and activates two forms of estrogen receptors, ERα and ERβ [22, 23]. To determinate if ERs play a key role in the regulation of the interested gene expression, we next examined the effect of ERα and ERβ on the estrogen-induced MLH1 expression. A real-time Q-PCR and Western blotting analysis showed that over-expression of ERβ increased the expression of *MLH1* at mRNA and protein level with estrogen, while ERα had no effect on the induction of the gene expression in LoVo cells (Figure 1C, D). Interestingly, we observed that E2 treatment failed to induce the *MLH1* expression when ERα was over-expressed or ERβ was knocked down (Figure 1C, D). To evaluate the function of endogenous Erβ in the regulation of MLH1 expression, we treated the cells with PPT, an ERα agonist, or DPN, an ERβ agonist. A Western blotting analysis indicated that the protein level of MLH1 was dramatically increased when the cells were treated with DPN, suggesting that the ERβ agonist boosted the gene expression via activation of ERβ (Figure 1E). Taken together, these results suggest that E2 prompted the expression of MLH1 mainly through ERβ but not ERα.

### Identification of the *MLH1* proximal promoter responsive to E2

**Figure 2:**
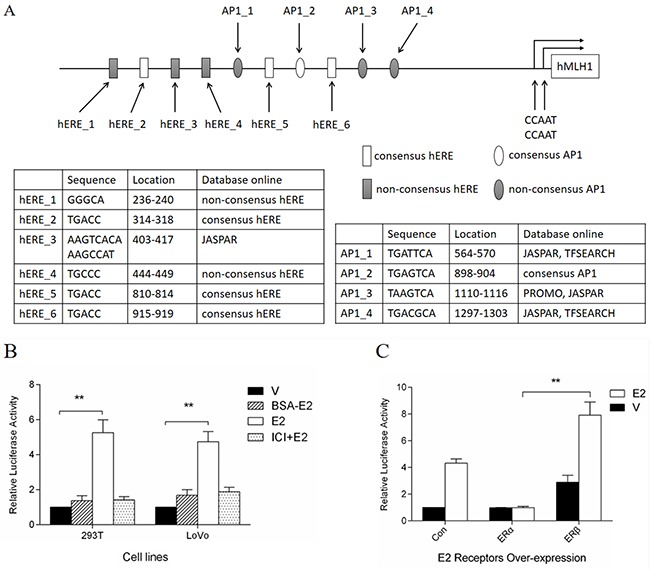
Dual-luciferase assay of *MLH1* promoter luciferase constructs **(A)** Schematic representation of *MLH1* proximal promoter. **(B)** Induction of E2 on luciferase activity. Hormone-depleted 293T and LoVo cells in 96-well plates were co-transfected with promoter luciferase construct pGL3-prom-luc and pRL-sv40. At 24 h post-transfection, cells were treated with 10 nM BSA-E2, E2 or vehicle, and 100 nM ICI 182.780 before E2 treatment in some groups. For another 24 h cells were lysed for luciferase assay. Values were normalized as previous description and represented as mean ± S.D. (n=3). ** *p* < 0.01 related to vehicle group. **(C)** Effects of ERs on luciferase activity. LoVo cells were prepared as **(B)**. Then cells were co-transfected with pGL3-prom-luc, pRL-sv40 and empty, ERα or ERβ expression plasmids. At 24 h post-transfection, cells were treated with 10 nM E2 or vehicle. Luciferase assay was performed, and values were normalized and represented as mean ± S.D. (n=3). ** *p* < 0.01 related to ERα over-expression group.

ERs regulate target genes through direct and indirect interaction with DNA. To identify the E2 binding sites in the promoter of *MLH1*, we performed a bioinformatics analysis using software TRANSFAC (http://www.gene-regulation.com/pub/programs), JASPAR (http://jaspar.genereg.net), ALGGEN PROMO (http://alggen.lsi.upc.es/cgi-bin/promo_v3/promo/promoinit.cgi?dirDB=TF_8.3), and TFSEARCH (http://www.cbrc.jp/research/db/TFSEARCH). We found several potential half-EREs in the *MLH1* proximal promoter region (Figure 2A, square boxes). Interestingly, we also observed several Activator Protein 1 (AP1) binding sites downstream of the EREs in the *MLH1* proximal promoter region (Figure 2A, oval boxes). These potential binding sites are echoed the facts that AP1 functioned as a co-activator for estrogen to regulate several other gene expression [24–27]. Therefore, we cloned this fragment (2.1 kb) into promoterless luciferase plasmid pGL3-Basic to examine the effect of ERβ and AP1 on the MLH1 expression *in vitro*. A luciferase reporter analysis demonstrated that E2 stimulated the luciferase activity significantly both in 293T and LoVo cells (Figure 2B, open columns), however, BSA-E2 had a weak effect on the luciferase activity and ICI 182.780, an estrogen receptor antagonist, obviously blocked the effect of E2 on the luciferase activity (Figure 2B). These results suggest that E2 increases the promoter activity of *MLH1* via canonical estrogen receptor pathway during the promotion of the gene expression.

Additionally, in order to verify the induction of MLH1 by E2 is mediated via ERβ not ERα, the reconstructed luciferase reporter plasmid, pGL3-prom-luc, was co-transfected with ERα or ERβ expression plasmids into LoVo cells, followed by stimulation with E2. Luciferase reporter analysis indicated that over-expression of ERβ, plus E2 treatment, increased luciferase activity significantly compared to control group (Figure 2C, third group). Consistent with previous findings, ERα over-expression, with or without E2 stimulation, failed to enhance the transcription of downstream genes of the interested promoter fragment (Figure 2C, second group). These results prove once more that E2 induces MLH1 expression mediated by ERβ, but not ERα.

### Half-EREs and AP1 binding sites are critical for the E2-induced *MLH1* gene expression

**Figure 3:**
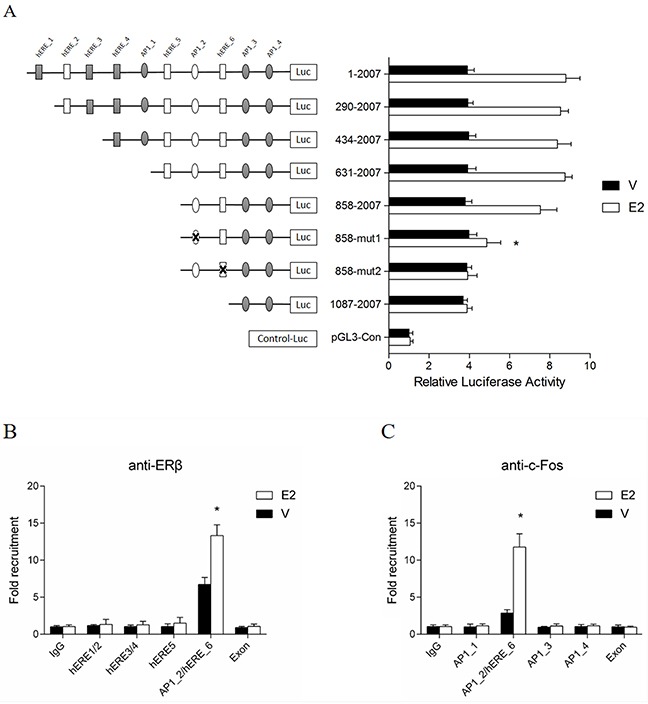
half-EREs and AP1 binding sites involved in the induction of E2 **(A)** Deletion and mutation analysis of *MLH1* promoter luciferase constructs. A series of luciferase constructs were transfected into hormone-depleted LoVo cells in 96 wells plate. 24 h later, cells were stimulated with vehicle or 10 nM E2 for 24 h, then lysed for luciferase assay. **(B & C)** ChIP assays of the binding of ERβ and AP1 to the *MLH1* promoter. Hormone-depleted LoVo cells in 100 mm^2^ dish were transfected with ERβ expression plasmid. After 24 h, cells were stimulated with 10 nM E2 or vehicle. ChIP samples were analyzed using specific antibodies against ERβ (B) and c-Fos (C). Immunoprecipitated DNA fragments and input DNA were analyzed by Q-PCR. *p < 0.01 compared with IgG control.

To determine which elements within the promoter region are responsive to E2, a series of deletion and mutation of the *MLH1* proximal promoter were generated to link to a luciferase reporter gene (Figure 3A, left panels). A luciferase reporter analysis for the functional transcription activity indicated that E2 remained response to the reporter when the fragments were deleted to 858 bps. Interestingly, the reporter activities were almost totally abolished when a ERE binding element and/or an AP1 binding site within the 858 region was mutated (Figure 3A, right panels). These results suggest that both of the ERE and AP1 binding sites in the 858 region of the promoter are critical for the E2-induced gene transcription.

Furthermore, to examine whether ERβ and/or AP1 were/was recruited to the specific elements within this *MLH1* proximal promoter, ChIP analyses were performed. Figure 3B showed that ERβ abundantly occupied the fragment containing AP1_2/hERE_6 (Figure 3B, black columns) while E2 greatly enriched the occupancy of ERβ in the binding site as precipitated by an anti-ERβ antibody (Figure 3B, open columns). Furthermore, we characterized the occupancy of AP1 in this fragment by a ChIP analysis. The result showed that c-Fos, a transcriptional factor binding to the AP1 site, occupied at AP1_2/ERE_6 region (Figure 3C, black columns) and E2 significantly increased the binding of c-Fos in the fragment containing AP1_2/hERE_6 (Figure 3C, open columns). These results suggest that, hERE_6 and AP1_2 (TGAGTCAGGTTGATTATGGTCA) in this proximal promoter are important for the MLH1 expression induced by E2.

### E2 promotes DNA mismatch repair and sensitizes CRC cells to 5-FU via induction of MLH1 expression *in vitro*

**Figure 4:**
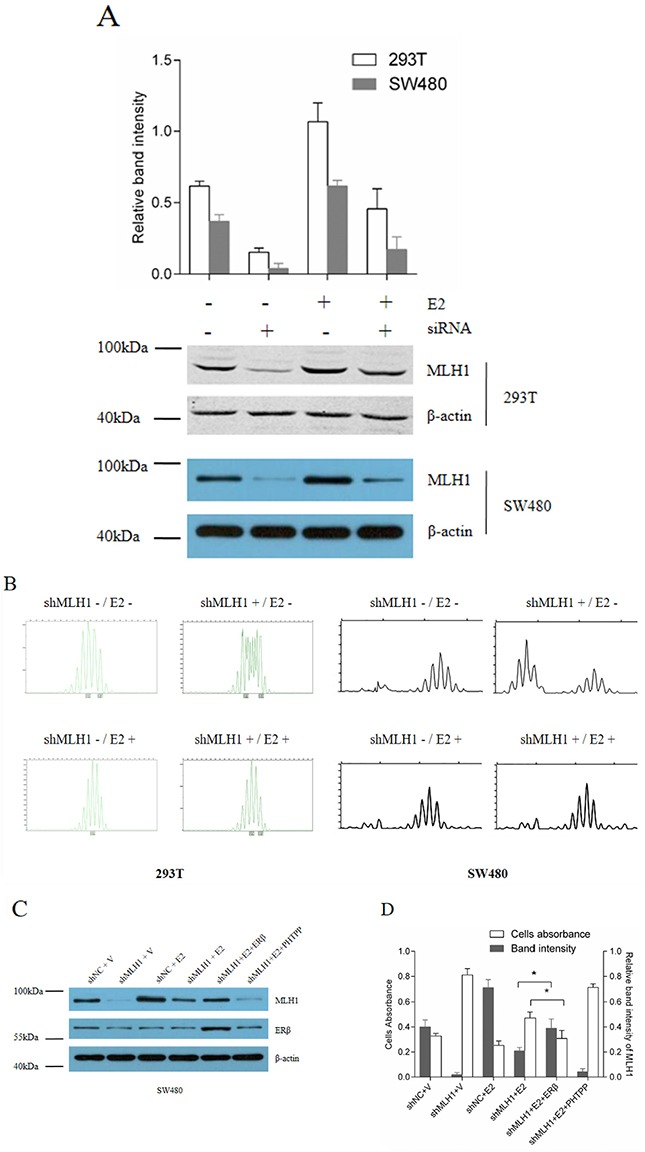
E2 promotes MMR via induction of MLH1 expression *in vitro* **(A)** Western blotting of endogenous MLH1 protein. Hormone-depleted 293T and SW480 cells in six-well plates were transfected with pGPU6/GFP/Neo-shMLH1 or negative control pGPU6/GFP/Neo-NC. At 24 h post-transfection, cells were treated with 10 nM E2 or vehicle for 48 h, then total protein were extracted and analyzed by Western blotting. **(B)** Microsatellite instability of 293T cell. Cells were transfected as part A. At 72 h post-transfection, total DNA were extracted, and MSI were analyzed by capillary electrophoresis. **(C)** Protein expression assay. SW480 cell lines were seeded in 6-well plates and divided into 6 groups, then each group was transfected with shNC (negative control), shMLH1 plasmid, or shMLH1 combined with ERβ expression plasmid. At 6 h post-transfection, cells were treated with vehicle, 10 nM E2 or 10 nM E2 plus 1 μM PHTPP. After 6 h, cells in every group were treated with 100 μM 5-FU and incubated for another 12 h. MLH1 and ERβ proteins were analyzed by Western blotting. **(D)** Cells viability and MLH1 expression level. SW480 cells were treated as indication in part A. Then cells viability were detected by CCK-8 kit, Relative band identity of MLH1 was shown as gray group. (* *p* < 0.05).

As reported in our previous studies, the expression of MLH1 is positively correlated with the concentration of E2 in a certain range [18, 19]. To examine the possibility that E2 promotes DNA mismatch repair ability through induction of MLH1 expression *in vitro*, endogenous MLH1 protein was depleted by transfection with shMLH1. As shown in Figure 4A, endogenous MLH1 protein was obviously decreased by the siRNA targeting *MLH1* gene in 293T and SW480 cell lines and treatment with E2 partially compensated the knockdown of MLH1 expression. Furthermore, the microsatellite instability (MSI) was observed at BAT-26 in cells with transfection of shMLH1 (Figure 4B). Interestingly, treatment with E2 dramatically decreased the MSI at the BAT-26 loci in the cells. These results suggest that E2 up-regulated MLH1 expression and enhanced MMR in cells.

In order to confirm that E2 enhance cells MMR ability by induction of MLH1 via ERβ, SW480 cells were transfected with shMLH1 alone or combination with ERβ expression plasmid, then incubated with vehicle, E2 or E2 combination with PHTPP, an ERβ antagonist, respectively, then treated with 5-FU. Protein expressions were analyzed by Western blotting (Figure 4C). At the same time, cells viability were determined by CCK8 assay. Results indicated that cells with insufficient MLH1 were insensitive to 5-FU (Figure 4D, second group). Whereas, over-expression of ERβ plus E2 treatment significantly reversed the influence of shMLH1 on cells sensitivity to 5-FU, through induction of MLH1 (Figure 4D, fifth group). But, E2 plus PHTPP, hardly increased the sensitivity of cells to 5-FU (Figure 4D, sixth group). These results support that E2 enhanced cells MMR through induction of MLH1 mediated by ERβ.

### ERβ agonist treatment induces MLH1 expression and inhibits tumor proliferation combined with 5-FU treatment in mice

**Figure 5:**
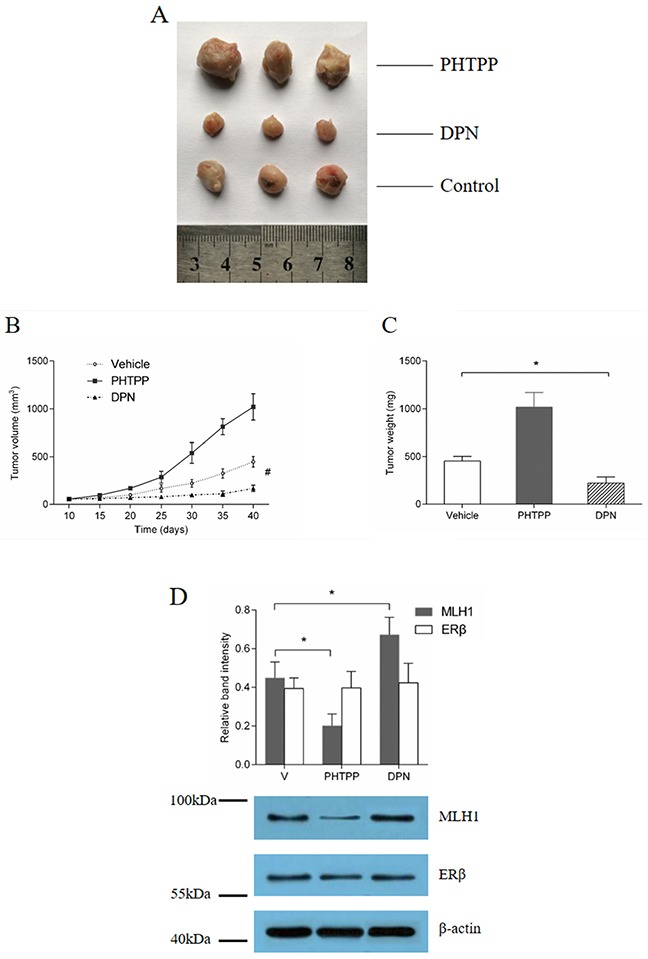
ERβ agonist inhibition of tumor growth *in vivo*, under treatment of 5-FU **(A)** tumor volume and **(B)** weight assay. Ovariectomized mice had HT29 cells implanted subcutaneously, and received treatment with DPN, PHTPP or Vehicle, combined with 5-FU respectively. Tumor size was observed every 5 days. **(C)** Protein expression analysis. Western blotting was used to analyze MLH1 and ERβ expression in the xenografts of each group. # *p* < 0.01 compared with control group. * *p* < 0.05 compared with control group.

On the 40 days after first injection, all those mice were sacrificed by cervical vertebra dislocation. Then xenografts were weighed and dissected (Figure 5A). We found that, under the treatment of 5-FU, the average size and weight of xenografts in DPN-treated group were significantly smaller than that of control group and PHTPP-treated group (# *p* < 0.01, * *p* < 0.05) (Figure 5B, C). Interestingly, significantly increases in the average volume and weight were observed in the PHTPP-treated group compared to control. The protein expressions assay demonstrated MLH1 expression decreased in the PHTPP-treated group, but increased in the DPN-treated group, compared with control (Figure 5D). Thus, we conclude that the ERβ agonist treatment increased MLH1 expression, which enhanced the sensitivity of CRC tumor to 5-FU and subsequently inhibited tumor proliferation *in vivo*.

## DISCUSSION

Previous studies indicated that females of a Lynch syndrome family with MMR gene(s) mutations were less likely to suffer from colorectal cancers than males with the same mutations [17, 28]. Epidemiological investigations supported E2 to be protective against colorectal cancer. Our group found that, there was a positive correlation between E2 level and MLH1 protein expression in normal colonic epithelia cells, but little relationship between E2 and MSH2. And *in vitro* findings showed that E2 enhanced MLH1 expression in colonic cells, but hardly affected MSH2 [19]. It implied that E2 induction of MLH1 expression may be one of the mechanism that E2 prevents against colorectal cancer. At present, most studies concerning E2 signal pathway in the CRC development focused on proliferation and apoptosis of cancer cells [29–32], however, we firstly study the correlation of E2 signal pathway and MLH1 in the mechanism of E2 prevention from CRC.

The present study demonstrates that E2 enhances MLH1 expression through estrogen receptors pathway, especially ERβ but not ERα. As shown in Figure 1A, because of large molecular weight, BSA-E2 cannot infiltrate into cells membrane or nuclear membrane. It typically binds estrogen receptors on cells membrane, such as G-protein coupled estrogen receptors, but hardly affects the nuclear receptors, ERα and ERβ for example. Besides, our results demonstrated that ICI 182.780 can block the effect of E2, and ERβ agonist significantly increased MLH1 expression. So, it can be concluded that E2 enhances MLH1 expression through estrogen receptors pathway, especially ERβ.

It has been reported that [33] ERα and ERβ are highly homologous in their DNA-binding domains (97% identity), but quite different in their transcription activation function regions (~ 20% identity). Consistently, we found in this study that over-expression of ERβ plus E2 stimulation activated *MLH1* transcription significantly, but there was little influence of ERα over-expression. Additionally, depletion of ERα moderately increased the expression of MLH1. These findings imply that over-expression of ERα could over-come the function of ERβ. On the other hand, depletion of ERβ almost invalidates the induction effect of E2 on *MLH1*. Interestingly, luciferase and ChIP assay suggest that, not only ERβ, but AP1 also play an important role in E2 induction of MLH1 expression. To our knowledge, many estrogen response genes don't have EREs or estrogen related transcription factor binding sites in their proximal promoter. Whereas, it's demonstrated that for 9 of 10 genes having ERβ binding sites in their proximal promoters, and 3 of 10 genes having ERβ binding sites in their enhancers were regulated by ERβ [34]. Additionally, the same researchers also reported that only 5% of ERβ binding regions include only ERE or half-ERE sites, however, approximately 60% of ERβ binding regions contain AP1 reactive elements together with ERE-like sites [34].

Our results further indicate that DNA MMR ability are increased by E2 through induction of MLH1 expression *in vitro*. MLH1 is one of the indispensable protein of MMR. It has been demonstrated that colorectal cancer patients whose tumors retain DNA MMR ability benefit from 5-florouracil (5-FU)-based chemotherapy, but those whose tumors lost MMR ability do not [35, 36]. Herein, our results show that ERβ plus E2 sensitize CRC cells to 5-FU through induction of MLH1 *in vitro*. Numerous reports supported that liganded ERβ was usually observed to inhibit proliferation, induce apoptosis, and to prevent from tumor development [32, 37–43]. Our findings confirm that ERβ agonist combined with 5-FU take an inhibitory effect on colorectal tumor proliferation *in vivo*. It's notable that, MLH1 expression levels is inversely correlated with volume and weight of xenografts under treatment of 5-FU. It means that liganded ERβ-induced MLH1 expression maybe sensitizes tumor cells to 5-FU chemotherapy.

## MATERIALS AND METHODS

### Chemicals

17β-estradiol (E2) (No. E2257), β-Estradiol 6-(O-carboxymethyl)oxime:BSA (BSA-E2) (No. E5630), 5-fluorouracil (5-FU) (No. 03738) were purchased from Sigma-Adrich and dissolved in 100% ethanol and PBS respectively. ERβ agonist Diarylpropionitrile (DPN) (No. 1494), ERα agonist propyl-pyrazole-triol (PPT) (No. 1426), ICI 182.780 (No. 1047) and ERβ antagonist 4-[2-phenyl-5,7-bis (tri-fluoro-methyl) pyrazolo [1,5-a] pyrimidin-3-yl] phenol (PHTPP) (No. 2662) were purchased from Tocris and dissolved in 100% ethanol. Transfection reagent FuGENE® HD (No. 4709705001) were purchased from Roche. The antibodies used are mouse anti-MLH1 (PAB11885, Abnova), mouse anti-ERα (ab32063, Abcam), mouse anti-ERβ (ab92306, Abcam), rabbit anti-c-Fos (ab27793, Abcam), mouse anti-β-actin (60008-1-Ig, Proteintech).

### Cells

293T cell lines, HT29, SW480 and LoVo colorectal cancer cell lines were purchased from Chinese Academy of Medical Sciences & Peking Union Medical College (PUMC). Cells were maintained in phenol red-free Dulbecco's modified Eagle medium (DMEM, Hyclone, USA) supplemented with 10% fetal bovine serum (FBS, Hyclone, USA). Cells were grown at 37°C in a humidified atmosphere of 95% air/5% CO_2_ and fed every two to three days. Before treatment, cells were washed with phosphate-buffered saline and cultured in DMEM with 1% charcoal-dextran stripped FBS (CD-FBS, Hyclone, USA) for 24 h to eliminate any estrogenic source, known as hormone-depleted.

### RNA interference

siRNA targeting *MLH1* (pGPU6/GFP/Neo-shMLH1) and negative control siRNA (pGPU6/GFP/Neo-NC) were purchased from GenePharma Co., Ltd (Shanghai, China). siRNA targeting *ERβ* (siERβ, HSS103380) and siRNA targeting *ERα* (siERα, HSS103377) were purchased from Life Technologies (Beijing, China). siRNA transfection were carried out using FuGENE® HD transfection reagent according to manufacturer's recommendations.

### Plasmid & constructs

The MLH1, ERα and ERβ expression plasmids were kindly provided by Chinese National Human Genome Center (Beijing, China). The luciferase reporter plasmid pGL3-Basic, pGL3-Control and pRL-SV40 vectors were purchased from Promega. The promoterless pGL3-Basic was used as the cloning backbone for the luciferase reporter plasmid in this study. The *MLH1* promoter (−1953 to +53) were amplified from the genomic DNA of human and cloned into pGL3-Basic, named pGL3-prom-luc. A series of truncations, containing -1664/+53 (290-2007), -1520/+53 (434-2007), -1323/+53 (631-2007), -1096/+53 (858-2007), -865/+53 (1087-2007) fragments of *MLH1* promoter, were generated by PCR. Two mutations, e.g. -1096/+53 (858-mut1) and -1096/+53 (858-mut2) were generated by site-directed mutagenesis using the QuickChange mutagenesis kit (Stratagene). AP1_2 TGAGTCA was mutated to TGCACCA. hERE_6 TGACC was mutated to TGGTC. Primers are listed in (Supplementary Table 2). All constructs were verified by sequencing.

### Luciferase assay

Cells were seeded on 24- or 96-well cell culture plates (Corning). Transient transfection was performed with FuGene HD (Roche) according to the manufacturer's instructions in DMEM with 1% CD-FBS overnight. On the next day, cells were treated with vehicle, ICI182.780, BSA-E2 or E2 for 24 h and then lysed in Passive Lysis Buffer (Promega). Luciferase activity was determined using Dual-Luciferase^®^ Reporter Assay System (Promega) and presented as normalized relative activity $\left(\frac{\text{sample}(\text{Firefly}/\text{Renilla})}{\text{control}(\text{Firefly}/\text{Renilla})}\right)$. Each luciferase assay was performed in triplicate, and all transfection experiments were repeated three or more times.

### Real-time quantitative PCR

Total RNA was extracted from cells using Trizol reagent (Invitrogen) according to the manufacturer's instruction and reversely transcribed with RevertAid™ First Strand cDNA Synthesis Kit (Fermantas). The protein- and DNA-free RNA was analyzed using iQ SYBR® Green Supermix (BIO-RAD) and specific primers are listed in (Supplementary Table 3). A final volume of reaction was kept in 10 uL. Real time Q-PCR was carried out on a Bio-Rad^®^ iQ^TM^5 Multicolor Real-time PCR Detection System (BIO-RAD). The amplification data was analyzed by iQ^TM^5 optical system software version 2.1. The relative gene expression was calculated with ΔΔC_T_ method.

### Western blotting

Total protein was extracted in lysis buffer with protease inhibitor cocktail (Sigma) on ice for 1 h. Protein was resolved by SDS-PAGE and transferred to PVDF membrane with iBlot^®^ Western Blotting System (Invitrogen) for 5 min. The membrane was incubated sequentially with primary antibody (Abnova) at 4°C overnight and secondary antibody combined with FITC at room temperature for 2 h. The membrane was scanned with Odyssey® Infrared Imaging System (LI-COR Biosciences). Each Western blotting experiment was performed in triplicate and a representative result is shown.

### Chromatin immunoprecipitation (ChIP)

ChIP experiments were performed on the basis of previously published protocols with minor modifications [20, 21]. Hormone depleted cells were grown in 10 cm dishes. After 24 h treatment with E2 (10^−8^M) or vehicle, cells were washed twice with cold PBS and then cross-linked with 1% formaldehyde for 10 min at 37°C. The cross-linking reaction was stopped by washing with 0.1 mol/L glycine. Samples were scraped, lysed and digested with Micrococcal Nuclease (ChIP Grade) to an average size of 200-1000 bps. The protein-DNA complexes were immunoprecipitated with antibodies as indicated. The antibody-protein-DNA complexes were adsorbed with protein A/G beads, then washed, eluted and were reversed overnight at 65°C. DNA was separated from protein by digestion with Proteinase K and analyzed by a real-time Q-PCR. The primers are listed in (Supplementary Table 4). Results are confirmed in two respective experiments.

### Determination of cells viability

Cells viability were analyzed with Cell Counting Kit-8 (CCK-8, Takara), according to the manufacturer's instructions. Cells were suspended in phenol-free DMEM and subsequently seeded in 96-well plates. After exposure to 5-FU, cells were incubated at 37°C for another 4 h with CCK-8 (10 μl per well). Absorbance was measured at a wavelength of 490 nm using GENios Pro microplate reader (Tecan, Männedorf, Switzerland).

### *In vivo* tumor growth inhibition study

All animal experiments were carried out in accordance with the guidelines issued by the Ethical Committee of Third Military Medical University. 4-5 weeks old female BALB/c nude mice, weighing 18-21 g, were anesthetized and bilaterally ovariectomized, and maintained in a pathogen-free environment. HT29 cells (5 × 10^6^) were subcutaneously injected into the ovariectomized mice. When xenografts reached 5 ± 1 mm, mice were divided into 3 groups with 5 mice in each group randomly. Each group were intraperitoneally injected with DPN (100 μl of 1 mg/kg per day), PHTPP (100 μl of 10 mM per day) or vehicle (100 μl per day), respectively. And each mouse was administered 5-FU (30 mg/kg) injection intraperitoneally, once weekly. Tumor size was monitored every five days by measuring the largest and smallest diameters of the tumor mass and estimated according to the following formula: volume=1/2×(largest diameter)×(smallest diameter)^2^. Finally, tumors were used to perform Western blotting analysis.

### Statistical analysis

Data are expressed as mean ± S.D. Statistical analysis was carried out by one-way ANOVA or Student's *t*-test, as appropriate. *P*-values < 0.05 were considered significant.

## SUPPLEMENTARY MATERIALS FIGURES AND TABLES



## Abbreviations

AP1: Activator protein1
BSA: Bovine serum albumin
ChIP: Chromatin immunoprecipitation
CRC: Colorectal cancer
DPN: Diarylpropionitrile
E2: Estradiol
ER: Estrogen report
ERE: Estrogen reponse elements
MMR: Mismatch repair
MSI: Microsatellite instability
PHTPP: 4-[2-phenyl-5,7-bis (tri-fluoro-methyl) pyrazolo [1,5-a] pyrimidin-3-yl] phenol
PPT: 1,3,5-Tris(4-hydroxyphenyl)-4-propyl-1H-pyrazole
