# Supplementary material for: Estrogen enhances mismatch repair by induction of MLH1 expression via estrogen receptor-β
Source: Oncotarget. 2017 Mar 18;8(24):38767–79. doi: 10.18632/oncotarget.16351 (PMC5503570; doi:10.18632/oncotarget.16351)
Supplement: Supplementary file 1 [file oncotarget-08-38767-s001.pdf]

Estrogen enhances mismatch repair by induction of MLH1 expression via estrogen receptor-β

SUPPLEMENTARY TABLES

Supplementary Table 1: Primers of RNA interference

Primers	Forward/Reverse	Sequences
pGPU6/GFP/Neo-shMLH1	Forward	GATCCGATCCGGTTCACTACTAGTAAACTTTCAAGAGA AGTTTACTAGTAGTGAACCTTTTTGGAAAGAAGAC
pGPU6/GFP/Neo-shMLH1	Reverse	GTCTTCAGCTTTTCCAAAAAAGGTTCACTACTAGTAAACT TCTCTTGAAAGTTTACTAGTAGTGAACCGG
pGPU6/GFP/Neo-NC	Forward	GATCCCACCGTTCTCCGAACGTGTCACGTCAAAGATTACG TGACACGTTCGGAGAATTTTTTGGAAGAC
pGPU6/GFP/Neo-NC	Reverse	GTCTTCGATCCAAAAAATTCTCCGAACGTGTCACGTAATC TCTTGACGTGACACGTTCGGAGAACG

Supplementary Table 2: Primers of truncations

Primers	Forward/Reverse	Sequences
Prom1F	Forward	CGGGGTACCTTAGTGTTTGCCTGAAGT
Prom290F	Forward	CGGGGTACCGCTTAACTTCGAAAGTACAA
Prom434F	Forward	CGGGGTACCGGACTGTTGTTGCCCCTA
Prom631F	Forward	CGGGGTACCATCCATTCGGATATGGTATTT
Prom858F	Forward	CGGGGTACCCTCTAGGATTGCCGACATG
Prom1087F	Forward	CGGGGTACCCTCTGCCTTGTGATA
Prom2007R	Reverse	CCGCTCGAGCCAGAAGAGCCAAGGA

Supplementary Table 3: Primers of real-time quantitative PCR

Primers	Forward/Reverse	Sequences
q_MLH1	Forward	GTGCTGGCAATCAAGGGACCC
q_MLH1	Reverse	CACGGTTGAGGCATTGGGTAG
q_GAPDH	Forward	GAGAAGGCTGGGGCTCATTT
q_GAPDH	Reverse	AGTGATGGCATGGACTGTGG

Supplementary Table 4: Primers of ChIP assay

Primers	Forward/Reverse	Sequences
AP1_2/ERE_6	Forward	AGGGACTCTAGGATTGCCGACA
AP1_2/ERE_6	Reverse	GACTTTAAAGCCTATTCTCTTGCCT
Exon	Forward	TGCATTTCTGCAGCCTCTGA
Exon	Reverse	CTGGGGTTGCTGGAAGTAGG
